# Detection of Expression Quantitative Trait Loci in Complex Mouse Crosses: Impact and Alleviation of Data Quality and Complex Population Substructure

**DOI:** 10.3389/fgene.2012.00157

**Published:** 2012-08-27

**Authors:** Ovidiu D. Iancu, Priscila Darakjian, Sunita Kawane, Daniel Bottomly, Robert Hitzemann, Shannon McWeeney

**Affiliations:** ^1^Department of Behavioral Neuroscience, Oregon Health and Science UniversityPortland, OR, USA; ^2^Oregon Clinical and Translational Research Institute, Oregon Health and Science UniversityPortland, OR, USA; ^3^Research Service, Veterans Affairs Medical CenterPortland, OR, USA; ^4^Department of Biostatistics-Public Health and Preventative Medicine, Oregon Health and Science UniversityPortland, OR, USA

**Keywords:** collaborative cross, eQTL detection, gene expression, mouse genetics, population substructure

## Abstract

Complex *Mus musculus* crosses, e.g., heterogeneous stock (HS), provide increased resolution for quantitative trait loci detection. However, increased genetic complexity challenges detection methods, with discordant results due to low data quality or complex genetic architecture. We quantified the impact of theses factors across three mouse crosses and two different detection methods, identifying procedures that greatly improve detection quality. Importantly, HS populations have complex genetic architectures not fully captured by the whole genome kinship matrix, calling for incorporating chromosome specific relatedness information. We analyze three increasingly complex crosses, using gene expression levels as quantitative traits. The three crosses were an F_2_ intercross, a HS formed by crossing four inbred strains (HS4), and a HS (HS-CC) derived from the eight lines found in the collaborative cross. Brain (striatum) gene expression and genotype data were obtained using the Illumina platform. We found large disparities between methods, with concordance varying as genetic complexity increased; this problem was more acute for probes with distant regulatory elements (*trans*). A suite of data filtering steps resulted in substantial increases in reproducibility. Genetic relatedness between samples generated overabundance of detected eQTLs; an adjustment procedure that includes the kinship matrix attenuates this problem. However, we find that relatedness between individuals is not evenly distributed across the genome; information from distinct chromosomes results in relatedness structure different from the whole genome kinship matrix. Shared polymorphisms from distinct chromosomes collectively affect expression levels, confounding eQTL detection. We suggest that considering chromosome specific relatedness can result in improved eQTL detection.

## Introduction

F_2_ intercrosses derived from standard inbred laboratory strains of mice and rats have been widely used to map complex trait quantitative trait loci (QTL). The design and implementation of such experiments is straightforward; further, over time there have been marked improvements in data analysis (e.g., Broman et al., 2003). However, this experimental design has two major drawbacks. One, the F_2_ crosses generally encompass only a small fraction of the genetic diversity available within *Mus musculus* or *Rattus norvegicus* and two, the mapping resolution, even with very large populations, is relatively poor (Darvasi et al., 1993). QTL mapping in heterogeneous stock (HS) populations addresses both of these issues. HS mice are derived by crossing multiple inbred strains (generally chosen for their genetic diversity), followed by outbreeding for numerous generations to increase the dimensions of the genetic map. The disadvantage of the extant HS populations is that each contains relatively few families (~50). Thus, family structure and genetic drift, are potentially confounding factors for data analysis. Plomin and McClearn (1993) appear to have been the first to suggest the use of murine HS populations for QTL analysis. Talbot et al. (1999), using HS/Ibg mice (formed by crossing eight inbred laboratory strains), were the first to demonstrate the marked increase in QTL mapping resolution. Demarest et al. (2001), using HS/Npt mice (a somewhat different eight-way cross), confirmed this observation. Recently, we have reported the development of a new HS population (Iancu et al., 2010), derived from the eight inbred strains that have been used to form the Collaborative Cross (Churchill et al., 2004). Because of the inclusion of three wild-derived strains, the HS-CC is 3–4 times more genetically diverse than other HS populations (Roberts et al., 2007). Thus, one of the issues inherent in HS QTL mapping, i.e., accurately determining the underlying haplotype, is particularly relevant here. The current study was initiated to evaluate the best approach to mapping in complex HS populations. To focus on the complexity issue, we examined different analysis strategies across a breadth of genetic diversity. Three different populations were examined: (1) a F_2_ intercross derived from the C57/BL6J (B6) and DBA/2J (D2) inbred mouse strains; (2) the HS4 derived by crossing the B6 and D2 strains with the BALB/cJ and LP/J strains; (3) the HS-CC.

The phenotypes used in this study are brain gene expression data described previously (Iancu et al., 2010); such high throughput gene expression studies allow the quantification of expression levels for tens of thousands of genes. However, the large volume of data also require careful pre-processing and filtering if spurious results are to be avoided. A variety of experimental design and technical factors can influence the reliability of gene expression and eQTL detection (Churchill, 2002). Standard approaches and software have been developed for the analysis of expression data; our initial processing steps for the expression data closely followed the approach described by (Du et al., 2008). In addition to this standard processing pipeline, several additional pre-processing steps have been recently suggested. Specifically, the array version used here consists of two different physical strips. It has been shown (Shi et al., 2009) that normalization procedures that take into account the strip assignment of probes result in improvement in the quantification of the expression levels. Additionally, the presence of SNPs within the probe sequence has also been shown to affect the accuracy of detection (Walter et al., 2007). Outlier samples in microarray data have been shown to affect the downstream analysis in several recent studies (Pearson et al., 2003; Baty et al., 2008; Ernst et al., 2008; Shieh and Hung, 2009); here we apply an outlier removal procedure to both the microarray and genotype data. Finally, filtering the array probes based on the probability of detection beneficially affects the quality of the data (Archer and Reese, 2009).

One common approach to QTL analysis is to test for association between genotypes at individual markers and the phenotype of interest [single-marker (SM) analysis]. A publicly available method that implements a variant of SM analysis and is suitable for use in complex mouse crosses is the efficient mixed-model association (EMMA; Kang et al., 2008)^1^. One essential feature of this method is the ability to control for sample relatedness in a computationally efficient manner. A different approach for genetic mapping is to integrate information from several markers, estimate the probability of descent from each of the founder inbred populations and evaluate if there are significant phenotype differences between alleles inherited from the different progenitor strains (Mott et al., 2000). An implementation of this method is available in the HAPPY program^2^; one advantage of this algorithm is the ability to identify the ancient strain allele effects. The present study evaluates concordance between these two methods. In order to combine the distinct advantages of each method, we suggest a procedure for combining the two algorithms, resulting in a “joint model” (JM) approach that identifies ancestral allele effects and also controls for kinship structure.

Different methods of QTL detection can result in conflicting outcomes, based on different assumptions about possible population substructure and the techniques used to adjust for it. As a result, a number of algorithms addressing this issue have been introduced (Devlin et al., 2001; Pritchard and Donnelly, 2001; Kang et al., 2008; Valdar et al., 2009; Listgarten et al., 2010). In most of these studies the genetic relatedness between samples is treated as a single confounding factor; the shared genetic background is assumed to control the phenotype through the collective action of loci dispersed throughout the whole genome. However, much less explored is the fact that relatively large genomic regions (such as individual chromosomes or other sets of haplotype blocks) can also vary in similarity between individuals. Critically, this relatedness is not fully captured by the whole genome kinship matrix but nevertheless affects the expression levels of large sets of genes. In the present study we show that relatedness based on specific genomic regions is a serious confounding factor and that it can be effectively attenuated using the procedures analyzed in this study.

Our paper is organized as follows. First, we compare the concordance between the two methods across the three mouse crosses; this comparison is repeated after additional data pre-processing steps. A procedure combining both haplotype reconstruction and kinship structure correction is introduced and its results compared with the original algorithms. Second, we show that the kinship structure in the two complex crosses, both genome-wide and at individual chromosomes, displays a strong influence: probe correlation with kinship structures results in excess eQTL detection. Each of these steps reveals potential pitfalls in current QTL detection procedures; our goal is to identify an optimal strategy for mapping in complex populations.

## Materials and Methods

### Animals

#### Breeding the HS-CC mice

Males and females of the eight parental strains (B6, CAST, NOD, 129, NZO, PWK, A, and WSB) were obtained from Jackson Laboratory. The strains were randomly assigned a letter from A to H; the order of assignment is the order noted above. The goal of the breeding strategy described below was to create a small panel (32 families) of the HS-CC mice; for such a small panel, a completely balanced breeding design is not possible. At G1 the following reciprocal F_1_ hybrids were formed: A × B, B × A; C × D, D × C; E × F, F × E; G × H, H × G. At G2 the following reciprocal four-way crosses were formed: AB × CD, CD × AB; BA × DC, DC × BA; EF × GH, GH × EF; FE × HG, HG × FE. At G3, 32 unique eight-way cross families were formed: ABCD × EFGH, ×GHEF, ×FEHG, ×HGFE…HGFE × ABCD, ×CDAB, ×BADC, ×DCBA. Each family was bred in duplicate. Of the 64 matings, 61 had litters; the three matings without litters were CDAB × EFGH, EFGH × CDAB, and CDAB × HGFE. Thus, all 32 of the planned families were formed. Beginning with G4, the families were outbred using a circle breeding design – a male from family 1 was bred to a female from family 2 and so on; the breeding was randomized to maintain the maximum diversity of the maternal and paternal chromosomes. At G6, the colony was expanded to 48 families by breeding a male from family 1 to a female from family 17 and so on. At G12 one male and one female from each family was randomly chosen for striatal gene expression analysis.

#### Breeding the HS4 mice

Males and females of the four parental strains (B6, D2, C, and LP) were obtained from the Jackson Laboratory. At G1 the 12 possible reciprocal F_1_ were hybrids were formed, followed at G2 by forming the 48 possible reciprocal four-way crosses. The four-way crosses were then outbred following a similar design to that noted for the HS-CC. At G19 one male and one female from each family was randomly chosen for striatal gene expression analysis. Details of the sample preparation are found in (Malmanger et al., 2006). Based on RNA quality, on maximizing family diversity and on gender neutrality, 64 samples were chosen for gene expression analysis. High quality data were obtained for 54 samples.

#### Breeding the F_2_ mice

Male and female B6 and D2 mice were obtained from the Jackson Laboratory. The reciprocal F_1_ hybrids were formed, followed by the formation of the four possible reciprocal F_2_ hybrids. Eight males and females were randomly selected from each of the reciprocal crosses. From the 64 samples, high quality expression data were obtained for 56 samples.

### Gene expression data processing

Gene expression data were obtained from the striatum using the Illumina WG 6.1 array exactly as described by the manufacturer. Data were imported into R^3^ using the lumi package (Du et al., 2008). Samples that were more than two standard deviations away from the mean inter-array correlation (Oldham et al., 2008) were not used in this study. This procedure was repeated three times resulting in stabilization of IAC and reduction of the datasets from 94 to 87 samples (HS-CC), 60 to 56 samples in F_2_, and 54 to 47 samples in the HS4.

Strip-level quantile normalization (Shi et al., 2009) was performed using a modified version of the procedure available in the lumi package. We removed from the data any probe that overlapped with known SNPs in any of the founding populations, using the publically available Wellcome Trust Sanger Institute database of known polymorphisms^4^. Further removed from analysis was any probe unlikely to be reliably detected (McClintick and Edenberg, 2006; Archer and Reese, 2009), using the detection-Call procedure available in the lumi R package. Using a cutoff threshold of 0.01, all probes not expressed in at least a quarter of the samples were removed.

### Computation of genome-wide and local genetic distances

For computing genetic distances between genomic locations, each genome was encoded as a long vector with entries in the range 0, 1, 2 based on the allelic content at a specific marker. Differences between two genomes were computed using the “vegdist” function in the “vegan” R package^5^, using the “manhattan” option for the distance function; this resulted in a kinship matrix similar to the one used by EMMA. For computing genetic differences at specific intervals, an identical procedure was employed, but using only the allelic differences at the two intervals flanking an interval. Our general procedure is to express pairwise relationships between individuals as square symmetric matrices, following the approach introduced in (Excoffier et al., 1992). For computing phenotype distances between individuals, the absolute value difference was used a distance measure.

## Results

### Initial comparison of HAPPY and EMMA results

**Figure 1:**
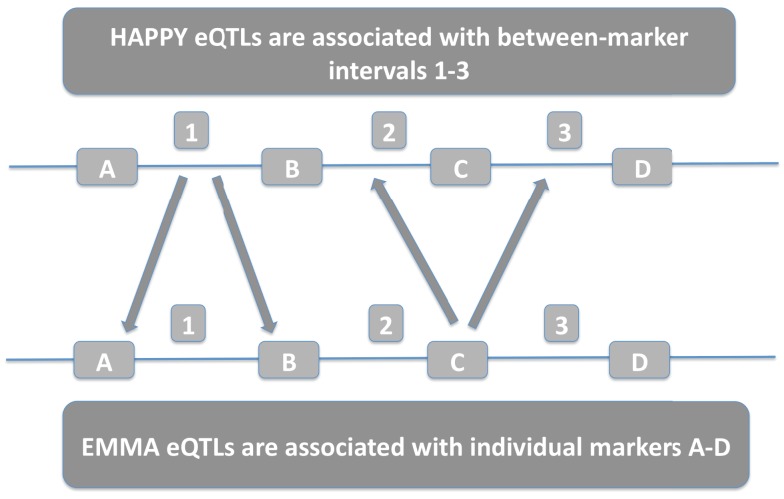
**HAPPY intervals are compared with EMMA individual markers**. HAPPY eQTLs associated with interval 1 correspond to EMMA eQTLs associated with either marker A or marker B. EMMA eQTLs associated with marker C correspond with HAPPY eQTLs associated with either interval 2 or 3.

The two detection methods differ in the way they quantify genetic variability. HAPPY evaluates whole genomic intervals between two successive markers while EMMA evaluates each marker independently. For our purposes, an interval that shows significant association in HAPPY was deemed to reproduce EMMA results if at least one of the flanking markers were associated by EMMA with the same gene expression level. The comparison procedure is summarized in Figure 1.

Genetic analysis is often performed in relation to one phenotype; the genomic location showing significant relationship is denoted as a QTL. In contrast, eQTL analysis considers the expression level of many genes as phenotypes of interest. In our study, we denote an eQTL as a significant relationship between a genomic location and a gene expression level. A gene could be modulated by several genomic locations while a genomic location could modulate several genes; here each of these pairs is regarded as a separate eQTL. Gene expression levels regulated by genomic locations in the immediate vicinity are denoted as *cis*, while genomic locations on different chromosomes or in distant locations on the same chromosome are denoted as *trans*. The exact delineation between *cis* and *trans* varies in the literature; in the present study we use the available marker density to determine the choice. For HAPPY, we consider a gene to be *cis* regulated if the gene is situated between the two genetic markers defining the genomic interval that modulates the gene. For EMMA, we define a regulated gene as *cis* if the significant marker is either of the two genetic markers flanking the gene.

In order to quantify the level of agreement between the methods, we employed an adapted version of Receiver Operator Characteristic (ROC) analysis (Huson et al., 1966). We kept the EMMA significance threshold fixed at *p* = 0.01 and we varied the HAPPY detection threshold between 0 and 1, recording the proportion of “false positives” (FP) and “true positives” (TP), based on the proportion of EMMA results recovered. The results span a curve within the unit square between the points (0,0) and (1,1), with chance overlap results falling on the main diagonal and perfect overlap including the (0,1) point; better overlap is denoted by the ROC curve “bending” more toward the (0,1) point and away from the main diagonal. Intuitively, more of a “bend” signifies that the TP are increasing faster than the false negatives as the threshold is increased.

**Figure 2:**
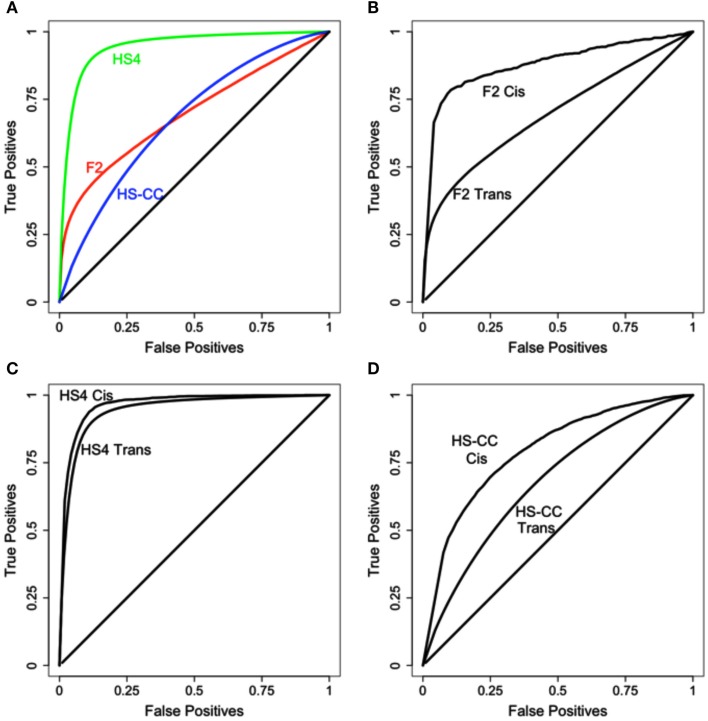
**Initial comparison of eQTL detection across mouse crosses and methods**. **(A)** Level of overlap across the three mouse crosses; HS4 results are superior to both F_2_ and HS-CC. **(B–D)** *Cis* results are more reproducible in all three datasets.

For the initial assessment of eQTL detection by HAPPY and EMMA gene expression was normalized and processed using the default options of the “lumiExpresso” function of the lumi R package (Du et al., 2008); no attempt was made to remove outlier samples or eliminate any array probes. The results are summarized in Figure 2; reproducibility varied with the genetic background (Figure 2A) with the best performance achieved in the HS4 data. Additionally, in all three cases better concordance was observed for the *cis* eQTLs (see Figures 2B–D).

### Impact of additional data pre-processing steps

In an attempt to improve the concordance between the two methods, a series of additional data pre-processing steps were performed. First, the gene expression samples were systematically examined for array outliers. The approach closely followed the strategy outlined in (Oldham et al., 2008). Briefly, correlations were calculated for each pair of samples across probe expression levels. For each sample, an average inter-array correlation was calculated; the distribution of these values was examined and any values that were two standard deviations from the mean were removed. This procedure was repeated three times resulting in stabilization of the inter-array correlation and reduction of the data sets from 94 to 87 samples (HS-CC), 60 to 56 samples F_2_), and 54 to 47 samples (HS4).

In an analogous manner the genotype data were examined for outliers. First, a kinship matrix was constructed for each of the populations in a manner similar to the procedure used by EMMA (Kang et al., 2008). The simplest form of kinship matrix was used, which assigns equal weight to each genomic location (markers) and adds all the allelic differences to arrive at a genome-wide measure of dissimilarity among individuals. The average distance between each individual to the population was computed and this distribution was examined for outliers, i.e., individuals with average distance more than two standard deviations from the mean. Four such outliers were found only in the HS-CC group; these samples were removed.

Recent work has demonstrated that the physical configuration of the Illumina WG 6.1 BeadChip array has an effect on array performance (Shi et al., 2009). Strip-level quantile normalization was performed using a modified version of the procedure available in the lumi R package. Next, data were culled for any probes that overlapped with known SNPs in any of the founding populations, using the publicly available Wellcome Trust Sanger Institute database of known polymorphisms^6^. Further removed from analysis was any probe unlikely to be reliably detected (McClintick and Edenberg, 2006; Archer and Reese, 2009), using the detection-Call procedure available in the lumi R package. Using a cutoff threshold of 0.01, all probes not expressed in at least a quarter of the samples were removed. Following these steps, the 45,000 probes represented on the Illumina WG 6.1 BeadChip array were reduced to 14825 probes (F_2_), 13758 probes (HS4), and 10233 probes (HS-CC).

**Figure 3:**
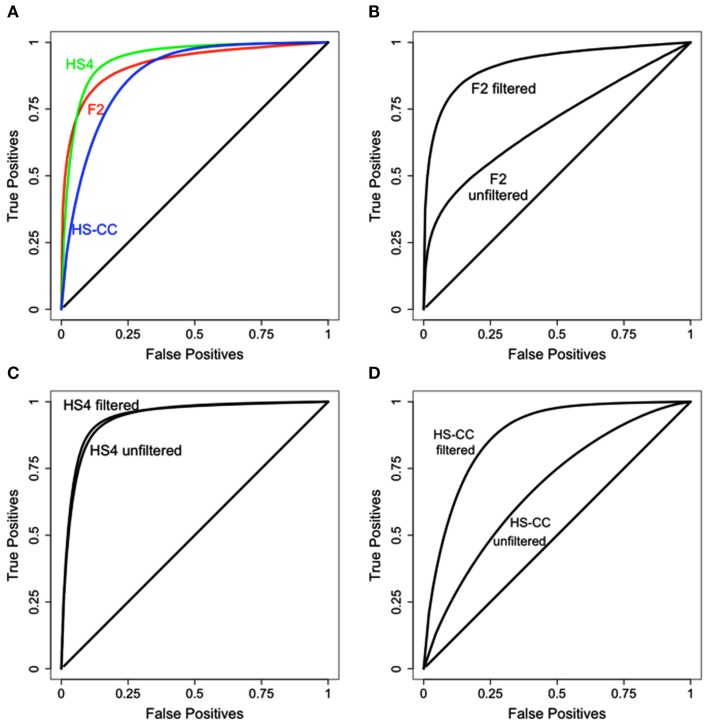
**Results of data filtering on the concordance between HAPPY and EMMA**. **(A)** Concordance comparison across the three data sets. HS4 concordance is best, with HS-CC and F_2_ slightly behind. **(B–D)** Concordance before and after data filtering. In all cases data filtering improves concordance between the methods.

The results of the data filtering steps outlined above are illustrated in Figure 3. Results from the HS4 data are still superior to the F_2_ and HS-CC, although to a much lesser extent compared to unfiltered data (see Figure 2A). Additionally, in each case data filtering results in a marked increase in concordance between methods (Figures 3B–D).

**Figure 4:**
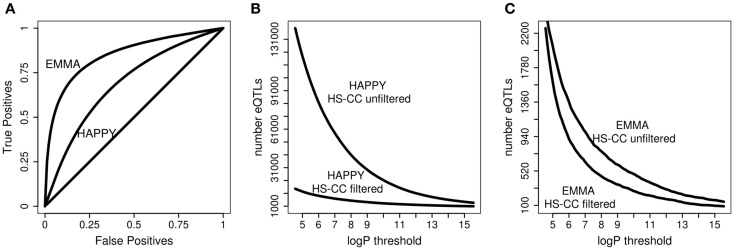
**Concordance of results and number of eQTLs for the probes retained after data filtering**. **(A)** The HAPPY and EMMA results for the retained probes are compared using ROC analysis. **(B,C)** Number of eQTLs detected by HAPPY and EMMA, respectively, before and after data filtering.

To evaluate the effects of data filtering on the number of eQTLs detected, we compared the concordance of eQTLs affecting only the probes left in the dataset after the filtering. In this comparison, the method and the identity of the probes were identical; the only difference was the data filtering. We observed that in the HS-CC data, the filtering steps resulted in relatively low concordance between the results, especially for the HAPPY procedure (Figure 4A). Additionally, we observed a large decrease number in the number of eQTLs detected, with HAPPY once again showing rather dramatic effects (Figures 4B,C). If the eQTLs detected in the unfiltered data are correct, then they should also be detected in the filtered data. The fact that they are not suggests that many of the eQTLs detected in the unfiltered data are in fact FP.

### Combining the HAPPY and EMMA detection procedures

In many cases, uncovering the founder strain origin of the allele generating an eQTL is of high interest. Population substructure can generate spurious associations between a locus and a gene expression level. Population stratification is manifested by a set of genomic locations that discriminate between the subpopulations, and also by a number of gene expression levels that are different between the same subpopulations. An eQTL detection procedure will likely detect associations between all genomic locations and expression levels that discriminate between the populations. These associations are questionable in light of the biological interpretation usually ascribed to eQTLs, which implies that genetic variations directly modulate the gene expression level. Including the sample relatedness information in the analysis can attenuate this problem by identifying the probe expression levels that are really caused with genome-wide differences between subpopulations, and not necessarily directly caused by a single eQTL that happens to be correlated with the genome-wide differences.

We attempted to retain both of these desirable properties by combining the two detection methods. We created the JM detection procedure by using the EMMA framework, but replacing the single-marker genotype information with the founder strain haplotype probability. This information is returned by the HMM inference procedure included in the HAPPY package.

**Figure 5:**
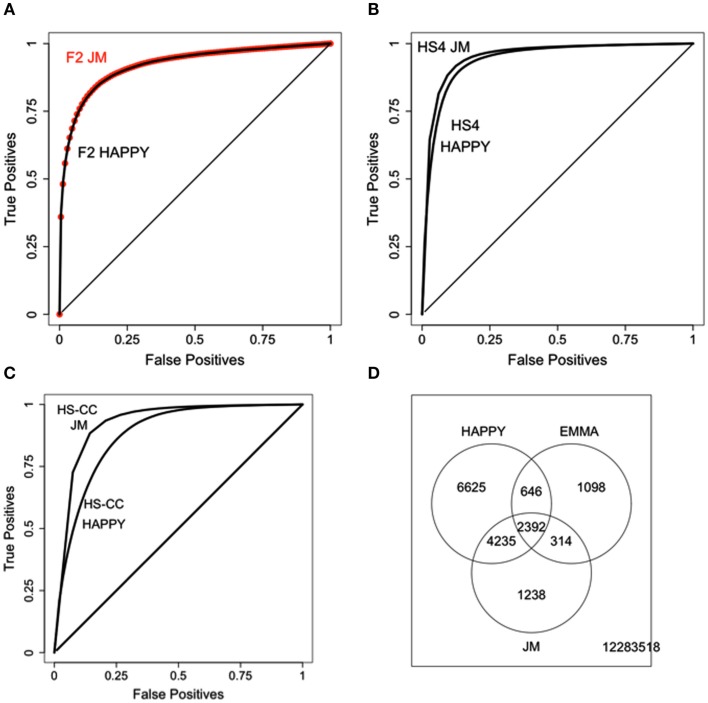
**Results of the JM procedure**. **(A)** JM compared with HAPPY in the F_2_ data. There is no improvement in the ability to reproduce the original EMMA results. **(B)** HS4 results show better ability of JM to reproduce EMMA results. **(C)** HS-CC results, JM has best improvement of JM of HAPPY. **(D)** Overlap of eQTLs (*p* < 10–5) across the three methods with JM detecting a large portion of intersection of HAPPY and EMMA results.

The results of the JM procedure are illustrated in Figure 5. In the F_2_ the sample relatedness is expected to be relatively uniform and, as expected, we do not observe an improvement of JM over HAPPY (see Figure 5A). In the HS4, even though the concordance between HAPPY and EMMA is already high, the JM still provides additional improvement (Figure 5B). The most substantial improvement is achieved in the HS-CC (Figure 5C). Overall, the JM procedure tends to recover probes in the intersection of the two original algorithms, as illustrated for the HS-CC (Figure 5D).

### Genetic architecture of F_2_ and HS crosses

While the genetic structure of the F_2_ population is well understood, the larger number of ancestral lines and breeding generations in the two HS populations can be expected to give rise to higher levels of genetic complexity.

As previously demonstrated for CD-1 mice (Aldinger et al., 2009), members of outbred populations are not necessarily equally related (or unrelated). A combination of constraints on the breeding schemes and allele fixation result in unpredictable correlations between the genetic structure of individuals; these correlations have implications for QTL detection (Valdar et al., 2009; Listgarten et al., 2010). In order to determine whether genetic relatedness was uniformly distributed across the genome, we computed the correlation between individual genomic intervals and the kinship matrix. Pairwise distances between individuals were computed using the genetic information at the two markers flanking an interval; these distances were arranged in a square symmetric matrix that was tested for correlation with the kinship matrix, using the Mantel test (Mantel, 1967). For the F_2_ data, 196 out of 213 genomic intervals available were correlated (*r* > 0.1) with the full genome kinship matrix, signifying that genetic relatedness is relatively uniformly distributed across the genome. However, for the HS4 and HS-CC data, only about half of the genomic intervals delineated by the available markers were correlated with the kinship matrix, implying that genetic relatedness is more varying across the genome.

The full genome kinship matrix is based on comparing the total number of alleles shared by individuals. An implicit assumption in this procedure is that genetic relatedness is uniformly distributed throughout the genome. Since this is not the case in the HS populations, we further explored this issue by constructing kinship matrices using the genetic information from individual chromosomes. If genetic relatedness were uniformly distributed, then the correlation between these chromosomal kinship matrices and the full genome kinship matrix would be uniformly high. However, we find that in the HS-CC these correlations are fairly low and uneven, varying between 0.32 for chromosome 18 and 0.48 for chromosome 2. Furthermore, when the chromosomal kinship matrices are correlated amongst themselves, we find that some pairs have correlation only as high as 0.27 (chromosomes 4 and 17) while other pairs have correlation as low as 0.01 (chromosomes 2 and 17). We conclude that kinship information is unevenly distributed amongst the chromosomes; for example while some samples are related according to the chromosome 2, they could appear unrelated according to chromosome 17. The situation is very similar in HS4, where correlations between chromosomal specific kinship matrices and the full genome kinship matrix range between 0.3 and 0.5, while the lowest correlation between chromosomal kinship matrices is −0.02.

### Kinship-probe correlations result in overabundance of eQTLs

In a similar manner to computing correlations between genetic relatedness matrices, a correlation value between the kinship matrix and an individual probe expression level can be computed. The absolute value difference in probe expression value between samples results in a square matrix; this matrix can then be correlated with the kinship matrix. In the HS-CC filtered dataset, of the 10,233 probes 279 probes displayed a correlation value >0.1 with the kinship matrix; slightly different thresholds did not substantially alter the results. We hypothesized that the 279 by 608 probe/interval pairs correlated with the kinship matrix will have an overabundance of eQTLs due to the kinship structure effects. In order to test this hypothesis, we computed the overlap between the selected marker/probe pairs and the total eQTLs detected by each method. Using the Fisher exact test, we found that the HAPPY results showed very significant overabundance of eQTLs for the selected probe/intervals (*p* < 2 × 10^−16^, odds ratio 6.6), where odds ratios above 1 denote overlap above chance. We report odds ratios as a more informative value since, for all comparisons, the Fisher test *p*-values were extremely low. We also verified that the number of eQTLs detected by HAPPY in each interval did not correlate with the length of the interval between the markers. For the EMMA and the JM procedures, the overabundance was still significant but at much lower levels (odds ratios of 1.62 and 1.32, respectively). This decrease in overabundance of eQTLs can be explained by the incorporation of the kinship information by the latter procedures. We find similar situation in HS4, where HAPPY results showed eQTL overabundance for selected interval/probe pairs (odds ratio 2.6), while overabundance was alleviated for EMMA and JM (odds ratios of 1.5 and 1.3, respectively). This analysis was not applicable in the F_2_, where almost all of genomic intervals were correlated with the kinship matrix.

The above results demonstrate that genetic differences dispersed throughout the whole genome collectively affect gene expression levels.

We subsequently inquired whether individual chromosomes show genetic differences that correlate with gene expression levels. In order to address this issue, we searched for probes that are correlated with the chromosome 1 kinship matrix (correlation >0.1) and we found 188 such probes. Additionally, we found 156 genomic intervals also correlated with the chromosome 1 kinship matrix. These combinations probe/intervals resulted in significant overabundance of detected eQTLs returned by all three methods, with odds ratios of 9.19, 3.89, and 3.02 for HAPPY, EMMA, and JM, respectively.

Importantly, we note that in spite of incorporating the full kinship matrix information, EMMA and JM did not fully eliminate the overabundance of eQTLs due to the shared genetic information on chromosome 1. The situation is similar for almost all 19 chromosomes: HAPPY results are highly overabundant for interval/probe pairs that are correlated with the chromosomal kinship matrix (odds ratios between 1.63 and 9.19, with mean odd ratio 4.44). EMMA and JM are still affected, albeit with less severity: for EMMA the odds ratios varied between 1.89 and 3.89 (mean 2.56), while for JM the odds ratios varied between 1.16 and 3.02 (mean 1.82). These values are highly significant, but they are smaller than the odds ratios for HAPPY, signifying that correction by the full genome kinship matrix has a beneficial effect. However, in order to control for the effects of shared genotypes on chromosome 1, the analysis has to include the chromosome 1 kinship matrix. We accomplished this adjustment using the chromosome 1 kinship matrix, incorporated into the EMMA analysis. As expected, this procedure significantly reduced the amount of overabundance of eQTLs: the odds ratio dropped from 3.89 to 2.01.

## Discussion

We performed two complementary comparisons of eQTL detection results: comparison across methods and a comparison across genetic backgrounds. The first comparison resulted in several steps that greatly improved detection reproducibility. The second comparison resulted in biological insights about the nature of genetic modulation of gene expression. We find that distributed genetic control of expression is detectable using genome-wide or chromosome-wide genetic relatedness.

Our results indicate that a naïve search of eQTLs in HS populations can result in large number of discordant results. We find that including low reliability data in the analysis results in low reproducibility across methods. We believe that results not reproducible across methods are more likely to be artifactual, and the fact that we can substantially increase reproducibility across methods by appropriately filtering the data strengthens this argument.

The eight founder inbred strains used in the development of the CC differ widely across a large set of phenotypes. When the same phenotypes are mapped in CC-derived populations, it is of interest to elucidate whether the effects of an ancient parental allele, now acting on a different genetic background, are concordant with the phenotype differences between the founder strains. Therefore, determining the likely origin of a genomic interval within CC-derived populations is desirable and HAPPY accomplishes this inference using an HMM approach. On the other hand, correcting for population kinship structure is also important, and EMMA accomplishes this in a computationally efficient manner, suitable even for the large datasets resulting from high throughput expression and genotyping studies. We retain both of these features in the JM procedure. As expected, JM preferentially detects eQTLs that were found by both HAPPY and EMMA. These results suggest that JM could an optimal approach for eQTL detection in complex crosses.

The full genome kinship structure of our HS populations is complex due to the specifics of the breeding process. A possible factor contributing to this uneven relatedness between samples is the relatively small number of breeding generations for our HS-CC population. The large differences in relatedness across chromosomes are surprising and their origin is unclear. We hypothesize that a possible explanation lies in the chromosome specific recombination dynamics. Recombination frequency is uneven across the genome, some of the inbred lines display differences in recombination rates and the rate of recombination is heritable (Koehler et al., 2002; Dumont and Payseur, 2011). A complex interplay between these factors could give rise to the chromosomal specific kinship structure observed here. The example of chromosome 1 is most illustrative. In the HS-CC HAPPY results, for the intervals/pairs correlated with the chromosome 1 kinship matrix, we detected an extreme level of eQTL overabundance, with odds ratio of 9.19. The overabundance was alleviated only partially by the EMMA and JM adjustment based on the full genome kinship matrix. This observation is interesting in light of a number of previous results. First, a region on distal chromosome 1 with strong influence on brain gene expression (eQTL hotspot) and neurobehavioral traits has been identified (Mozhui et al., 2008). Second, recombination on mouse chromosome 1 is dominated by “recombination hotspot,” regions of high recombination frequency (Kelmenson et al., 2005). In our HS populations, we observed that a significant number of distant genetic markers display relatively high levels of LD. The latter observations implies that long-range linkage within chromosome 1, partially due to localized recombination events, could generate a complex structure of relatedness across individuals that does not necessarily mirror the other chromosomes or the full genome kinship relationships. Also, the presence of recombination hotspots suggests that simply increasing the number of breeding generations will not resolve the issue of divergence of kinship structure across chromosomes. This possibility is supported by the fact that divergence was still present in the HS4 population, which had a larger numbers of generations compared to the HS-CC.

Regardless of the causes, the variability of the relatedness structure across the genome strongly affects eQTL detection. We observed significant overabundance of eQTLs for specific interval/probe pairs, which we were able to predict using the correlation structure between probes, genomic intervals, and kinship matrices. While the confounding effects of the full kinship matrix are attenuated by EMMA and JM procedures, the overabundance due to chromosomal specific kinship structure remains. A possible solution for this problem is the inclusion of additional kinship matrices in the analysis pipeline, as demonstrated by the HS-CC EMMA results on chromosome 1. However, the chromosome specific kinship information could be incorporated into any other methods that adjust for the full genome kinship structure.

These results have potential biological implications. First, we show that large numbers of probes are correlated with either the full genome kinship matrix or with specific chromosomal differences between the samples. This implies that significant biological modulation of gene expression is due to correlated genetic polymorphisms distributed across large genomic regions. This “distributed” modulation of biological activity likely coexists and interacts with the one-to-one relationship between genotype and phenotype that is the focus of QTL studies. One approach of uncovering this distributed genetic control is the matrix correlation technique employed in the current study. A second biological implication regards the effect of population substructure on genetic associations. While we show that chromosomal specific effects have the potential to confound interval/marker associations, we also note the converse: it is possible for individuals that are not closely related according to the full genome kinship matrix to have similar transcription profiles, provided that they are related according to one of the chromosomal kinship matrices.

## Conflict of Interest Statement

The authors declare that the research was conducted in the absence of any commercial or financial relationships that could be construed as a potential conflict of interest.

## Footnotes

^1^http://mouse.cs.ucla.edu/emma/

^2^http://www.well.ox.ac.uk/happy/happy1.2.shtml

^3^http://www.r-project.org

^4^http://www.sanger.ac.uk/resources/mouse/genomes/

^5^http://cran.r-project.org/web/packages/vegan/index.html

^6^http://www.sanger.ac.uk/resources/mouse/genomes/
